# Added value of regional ^18^F-FDG PET/MRI-assisted whole-body ^18^F-FDG PET/CT in malignant ascites with unknown primary origin

**DOI:** 10.1186/s41824-023-00179-0

**Published:** 2023-12-04

**Authors:** Yiru Fu, Weiwei Ruan, Xun Sun, Fan Hu, Xiaoli Lan, Fang Liu

**Affiliations:** 1 Department of Nuclear Medicine, Union Hospital, Tongji Medical College, Huazhong University of Science and Technology, Wuhan, 430022 Hubei China; 2 Hubei Key Laboratory of Molecular Imaging, Wuhan, 430022 Hubei China; 3 Key Laboratory of Biological Targeted Therapy of the Ministry of Education, Wuhan, 430022 China

**Keywords:** PET/MRI, PET/CT, ^18^F-FDG, Malignant ascites, Cancer

## Abstract

**Background:**

Comparing to PET/CT, integrative PET/MRI imaging provides superior soft tissue resolution. This study aims to evaluate the added value of regional delayed ^18^F-FDG PET/MRI-assisted whole-body ^18^F-FDG PET/CT in diagnosing malignant ascites patients.

**Results:**

The final diagnosis included 22 patients with ovarian cancer (n = 11), peritoneal cancer (n = 3), colon cancer (n = 2), liver cancer (n = 2), pancreatic cancer (n = 2), gastric cancer (n = 1), and fallopian tube cancer (n = 1). The diagnosis of the primary tumor using whole-body PET/CT was correct in 11 cases. Regional PET/MRI-assisted whole-body PET/CT diagnosis was correct in 18 cases, including 6 more cases of ovarian cancer and 1 more case of fallopian tube cancer. Among 4 cases that were not diagnosed correctly, 1 case had the primary tumor outside of the PET/MRI scan area, 2 cases were peritoneal cancer, and 1 case was colon cancer. The diagnostic accuracy of regional PET/MRI-assisted whole-body PET/CT was higher than PET/CT alone (81.8% vs. 50.0%, κ ^2^ = 5.14, p = 0.023). The primary tumor conspicuity score of PET/MRI was higher than PET/CT (3.67 ± 0.66 *vs*. 2.76 ± 0.94, P < 0.01). In the same scan area, more metastases were detected in PET/MRI than in PET/CT (156 vs. 86 in total, and 7.43 ± 5.17 *vs*. 4.10 ± 1.92 per patient, t = 3.89, P < 0.01). Lesion-to-background ratio in PET/MRI was higher than that in PET/CT (10.76 ± 5.16 *vs.* 6.56 ± 3.45, t = 13.02, P < 0.01).

**Conclusion:**

Comparing to whole-body PET/CT alone, additional delayed regional PET/MRI with high soft tissue resolution is helpful in diagnosing the location of the primary tumor and identifying more metastases in patients with malignant ascites. Yet larger sample size in multicenter and prospective clinical researches is still needed.

## Background

Malignant ascites is defined as abnormal accumulation of fluid in the peritoneal cavity due to a variety of malignant tumors (Becker et al. 2006). It accounts for about 10% of all ascites cases. It is known that about 50% of patients with malignant ascites present with ascites at the initial diagnosis (Saif et al. 2009), and up to 20% of patients with malignant ascites have tumors of unknown primary origin (Becker et al. 2006). For most patients, the development of malignant ascites indicates an advanced and incurable outcome (Ayantunde and Parsons 2007). However, for ovarian cancer, patients are responsive to systemic chemotherapy and/or locoregional treatment, and may have a long survival time (Ayantunde and Parsons 2007; Adam and Adam 2004; Kipps et al. 2013). Therefore, it is important to identify the primary origin of malignant ascites.

^18^F-FDG PET/CT has been widely used in tumor detection, which is helpful in identifying the primary cause of ascites (Zhang et al. 2009; Han et al. 2018). For metastatic cancer with unknown primary origin, 20–60% of primary tumors can be identified by ^18^F-FDG PET/CT (Nanni et al. 2005; Ambrosini et al. 2006; Podoloff 2009; Sekine et al. 2017). Comparing to PET/CT, integrative PET/MRI imaging provides superior soft tissue resolution, enabling more comprehensive assessment of soft tissue in abdomen and pelvis (Bagade et al. 2015; Xin et al. 2016; Galgano et al. 2021). Functional MRI sequences, such as diffusion-weighted imaging (DWI), can improve diagnostic efficiency (Ehman et al. 2017; Padhani et al. 2011). Previous studies have investigated the benefits of using PET/MRI in staging of primary abdominal and pelvic cancer, and in detecting metastasis in abdominal organs such as liver (Xin et al. 2016; Galgano et al. 2021). Clinical evidence for PET/MRI in patients with malignant ascites, however, is still lacking.

This study is to evaluate the added value of regional delayed ^18^F-FDG PET/MRI-assisted whole-body ^18^F-FDG PET/CT in diagnosing primary and metastatic lesions in patients with malignant ascites.

## Methods

### Patients

Retrospective analysis of 22 patients with malignant ascites who underwent ^18^F-FDG PET at our center from January 15, 2018 to June 30, 2022. Inclusion criteria: (1) Ascites cytology and/or tumor markers suggesting malignant ascites; (2) Unknown primary tumor prior to PET scan; (3) All patients underwent ^18^F-FDG PET/CT and regional PET/MRI examination. Exclusion criteria: (1) Blood glucose exceeding 12 mmol/L; (2) Previously diagnosed malignant tumor; (3) The diagnosis cannot be confirmed by follow-up.

This retrospective study was approved by the Ethics Committee of Union Hospital, Tongji Medical College, Huazhong University of Science and Technology. All patients have signed an informed consent before undergoing ^18^F-FDG PET/CT and PET/MRI examinations. There were no additional follow-up requirements in this study, and only clinical and imaging data of patients in our hospital were reviewed.

### ^18^F-FDG PET/CT protocol

Compound FDG was synthesized automatically after ^18^F was produced by a cyclotron (MINI trace, GE Healthcare), with a radiochemical purity greater than 95%. All patients fasted for at least 6 h before the administration of ^18^F-FDG. 3.7–4.4 MBq/kg of ^18^F-FDG was injected intravenously. Approximately 60 min after injection of ^18^F-FDG, patients were instructed to rest in a quiet room with minimal activity before undergoing PET/CT image acquisition. Imaging was performed using a Discovery VCT FDG PET/CT system (GE Healthcare, Milwaukee WI, United States). A low-dose CT scan was obtained with the following parameters: tube voltage of 120 kV, tube current of 80 mAs, and section collimation of 3.75 mm. A PET scan was then immediately acquired from top of the head to the upper part of the legs in 2D mode with 3 min per bed position. PET data were reconstructed with the ordered set expectation maximization algorithm, and attenuation was corrected by CT images. Data from both CT and PET were sent to a nuclear medicine workstation (Xeleris Workstation, GE Healthcare) for clinical evaluation. The standardized uptake value (SUV) of the lesions was calculated as tissue activity (expressed as megabecquerels per milliliter of tissue) divided by injected dose (expressed as megabecquerels per gram of body weight).

### ^18^F-FDG PET/MRI protocol

About 120 min after ^18^F-FDG PET/CT whole-body imaging, the patient underwent regional PET/MRI imaging. The scan area was determined as one or three PET beds (pelvis or whole abdomen) according to PET/CT images and abnormal items of tumor markers. The imaging was performed with a hybrid time-of-flight (TOF) PET/MRI (SIGMA™ PET/MRI, GE Healthcare, Waukesha, WI, USA). The duration for the PET is 7 min per bed with respiratory gating. The reconstruction parameters is as followed: FOV = 50 cm*50 cm, Filter Cutoff = 5.0 mm, Subsets = 28, Iterations = 2, Matrix = 192*192. MR sequences included T2-weighted imaging with fat suppression (fs T2WI, FOV = 48 cm*48 cm, slice thickness/ slice spacing = 5 mm/1 mm, Matrix = 384*384, time of echo = 78 ms, time of repetition was automatic based on the number of slices, ETL = 28), the diffusion-weighted imaging with fat suppression (fs DWI, FOV = 48 cm*40 cm, slice thickness/ slice spacing = 5 mm/1 mm, Matrix = 192*128, b value = 0 and 800 s/mm^2^), and the 3D liver acquisition volume acceleration T1-weighted imaging (3D-LAVA, Flip Angle = 12°, BW = 142.86 kHz).

### Image analysis

To avoid bias, two experienced nuclear medicine physicians (F.L. with 20 years of experience in radiology and 6 years of experience in nuclear oncology, X.S. with 25 years of experience in nuclear medicine) have independently analyzed the ^18^F-FDG PET/CT and regional PET/MRI images. Image interpretation consists of visual analysis and semi-quantitative assessment, and the results were discussed to reach a consensus.

For visual analysis, the conspicuity score for the diagnosis of primary tumors is based on a 4-point scoring system (1 point: no clear primary tumor is found; 2 points: multiple suspicious lesions are found, and the primary tumor cannot be determined; 3 points: possible primary tumor is found; 4 points: primary tumor is clear). The peritoneum, parenchymal organ, bone metastases, and metastatic lymph nodes in the common area scanned by PET/CT and PET/MRI were counted. For semi-quantitative assessment, the maximum standardized uptake value (SUVmax) was recorded in regions of interest (ROIs) drawn over the lesions identified by the nuclear medicine physicians using the nuclear medicine workstation (GE AW4.6 workstation software, GE Healthcare, Milwaukee, WI, USA). The SUVmax of both lesions and muscles was measured, and then, the lesion-to-background ratios were calculated.

### Reference standard and follow-up

A combination of biopsy, surgical pathological results, prior imaging findings, and clinical and imaging follow-up (median: 216.5 days, range: 26–1504 days) was used as the reference standard for the lesion. Histopathologic analysis of biopsy or surgery samples was used as the gold standard in determining the primary tumor identity, but it was not practical for all cases. In those circumstances, follow-up CT and/or MR images, a comprehensive analysis of follow-up examinations and elevated tumor markers in serum or ascites, were used to assess the identity of the primary tumor. For metastases, due to the presence of ascites, peritoneal and omentum thickness and/or nodules with higher ^18^F-FDG uptake than background were considered as metastases. Others were determined by surgical pathology, imaging malignant features, imaging follow-up progress, clinical follow-up progress, and whether cancer treatment was effective.

### Statistics

Statistical analysis was performed using R version 4.1.3 (2022-03-10). McNemar test was used to compare the diagnostic accuracy of primary tumor. Wilcoxon signed-rank test was used to compare the scores of primary tumors. Paired t test was used to compare the number of metastases and SUVmax ratio of lesion and muscle. For all analyses, P < 0.05 was considered statistically significant. The results were presented as the mean ± SD.

## Results

### Patient characteristics

The characteristics of the patients are presented in Table 1. A total of 22 patients (4 males and 18 females) with malignant ascites were included in this retrospective study. The average patient age was 61.73 ± 13.56 years (range: 32–92 years).

**Table 1:** Patient characteristics

Characteristics	No. (%) of patients
Age	
< 60	8 (36.4)
≥ 60	14 (63.6)
Sex	
Male	4 (18.2)
Female	18 (81.8)
Reference standard	
Histopathological examination	11 (50.0)
Follow-up	11 (50.0)
Type of primary tumor	
Ovarian cancer	11 (50.0)
Peritoneal cancer	3 (13.6)
Colon cancer	2 (9.1)
Liver cancer	2 (9.1)
Pancreatic cancer	2 (9.1)
Gastric cancer	1 (4.5)
Fallopian tube cancer	1 (4.5)

Before the PET examination, twenty patients underwent serum or ascites tumor marker tests including CA125, CA153, CA19-9, etc. Fifteen patients had multiple abnormal tumor markers in serum and ascites, while five patients had only one abnormal tumor marker. Two patients had no tumor marker information. Primary tumors were identified through biopsy and surgical pathological analysis in 11 cases, and metastases were identified through surgical pathological analysis in 8 cases. The final diagnosis of 22 patients included ovarian cancer, peritoneal cancer, colon cancer, liver cancer, pancreatic cancer, gastric cancer, and fallopian tube cancer.

### Diagnosis of primary origin

**Fig. 1:**
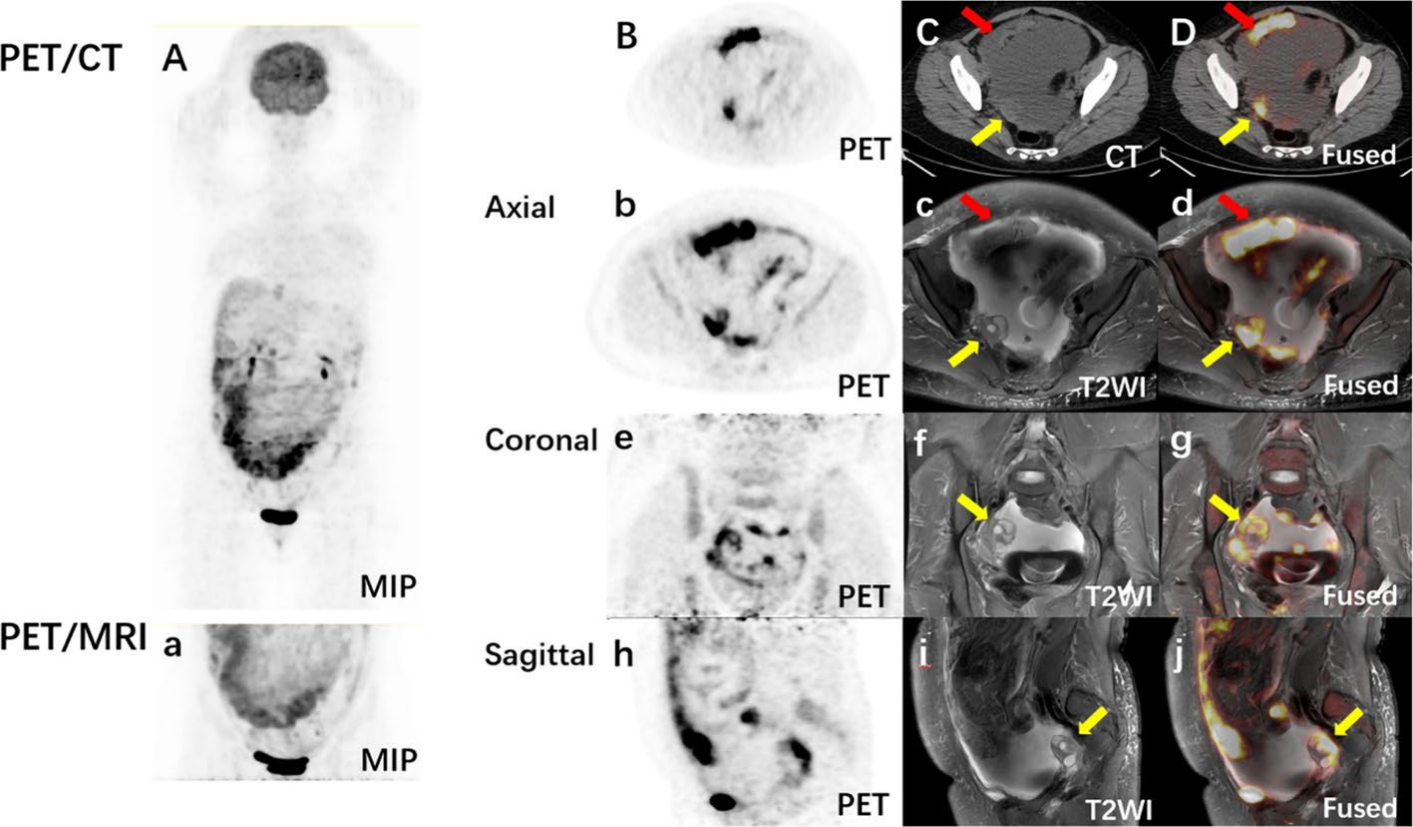
A 47-y-old woman with cancer cells detected in ascites and serum tumor marker CA125 greater than 5000U/ml. PET/CT images (**A–D**) show two focal increased FDG uptake in the pelvic cavity and corresponding slightly high dense lesions on the CT images (**C**, arrows). PET/MRI images (**a**–**j**) show higher FDG uptake. One lesion was solid (**c**, red arrow) and the other was cystic solid (**c**, **f**, **I**, yellow arrow) on T2-weighted images. The location and composition of the lesions showed by MRI help distinguish primary ovarian lesions (yellow arrow) from peritoneal metastases (red arrow)

The detailed diagnosis information for primary origin with PET/CT and PET/MRI is listed in Table 2. Among the 22 cases, the diagnosis of primary origin using whole-body PET/CT was correct in 11 cases. Among the other 11 cases, in which the primary tumor could not be identified by PET/CT, the majority were ovarian cancers (6 cases). Regional PET/MRI-assisted whole-body PET/CT has correctly diagnosed the primary origin in 18 cases. Among the 7 cases, in which PET/MRI diagnosed correctly while PET/CT misdiagnosed, 6 were ovarian cancers and 1 was fallopian tube cancer. In these cases, the structure and mixed solid and cystic components of ovaries were shown more clearly on MRI than on CT images, which was the key of improved the diagnosis (Fig. 1). In the remaining 4 cases that were not correctly diagnosed, 1 case had primary tumor outside of the PET/MRI scanning region, the other 2 cases of peritoneal cancer and 1 case of colon cancer been misdiagnosed as ovarian origin. The diagnostic accuracy of regional PET/MRI-assisted whole-body PET/CT was higher than that of PET/CT alone (81.8% vs. 50.0%, κ^2^ = 5.14, p = 0.023).

**Table 2:** Diagnosis of primary origin by whole-body PET/CT and regional PET/MRI-assisted whole-body PET/CT

Diagnosis	No. of patients	No. (%) of patients diagnosed correctly	
		PET/CT*	PET/MRI + PET/CT^#^
Ovarian cancer	11	5	11
Peritoneal cancer	3	0	c
Colon cancer	2	1	c
Liver cancer	2	2	2
Pancreatic cancer	2	2	2
Gastric cancer	1	1	1
Fallopian tube cancer	1	0	1
Total	22	11 (50.0)	18 (81.8)

*PET/CT is an abbreviation for whole-body PET/CT

^#^PET/MRI + PET/CT is an abbreviation for regional PET/MRI-assisted whole-body PET/CT

In terms of conspicuity score for the diagnosis of primary tumors, PET/MRI revealed a significantly higher conspicuity than PET/CT (3.67 ± 0.66 vs. 2.76 ± 0.94, P < 0.01).

### Diagnosis of metastases

**Fig. 2:**
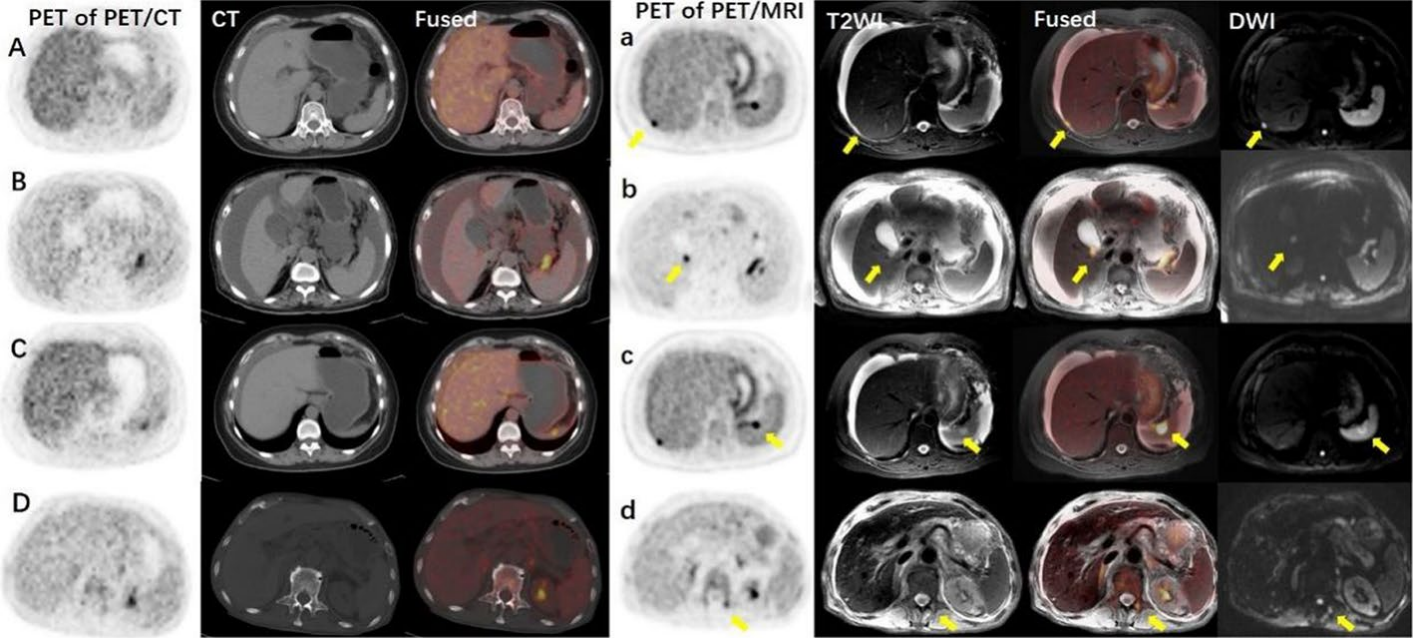
Small metastasis lesions (yellow arrows) detected by PET/MRI but missed by PET/CT. Line 1 (**A** and **a**) A 60-y-old woman with ovarian cancer. Peritoneal metastasis. Line 2 (**B** and **b**) A 75-y-old woman with ovarian cancer. Liver metastasis. Line 3 (**C** and **c**) Same case of Line 1. Spleen metastasis. Line 4 (**D** and **d**): A 65-y-old man with pancreatic cancer. Lumbar metastasis

**Fig. 3:**
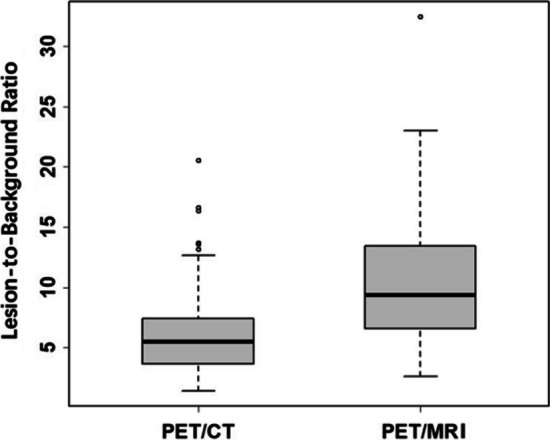
The lesion-to-background ratio of SUVmax in PET/CT and PET/MRI (boxplot). The box represents the upper and lower quartiles; the horizontal bar represents the median. The points represent extreme values. The lesion-to-background ratios of SUVmax in PET/MRI show higher than that in PET/CT

In the same scan range of both PET/MRI and PET/CT, PET/MRI detected 156 metastases, while PET/CT detected 86 metastases. For each patient, PET/MRI found more metastases than PET/CT (7.43 ± 5.17 vs. 4.10 ± 1.92, t = 3.89, P < 0.01; Table 3). The small metastasis lesions on T2WI and DWI were more obvious than on CT images. Four parenchymal organ metastases and two bone metastases lesions, in which having diameters from 4 to 7 mm, were detected only on T2WI and DWI (Fig. 2). For the metastases lesions found on both PET/CT and PET/MRI (n = 86), the lesion-to-background ratios of SUVmax on PET/MRI were higher than PET/CT (10.76 ± 5.16 vs. 6.56 ± 3.45, t = 13.02, P < 0.01, Fig. 3).

**Table 3:** Number of metastases identified by PET/CT and PET/MRI within the same scan range

Metastases	No. of lesions	
	PET/CT	PET/MRI
Peritoneal metastases	63	116
Parenchymal organ metastases	2	6
Bone metastases	1	3
Metastatic lymph nodes	20	31
Total	86	156

## Discussion

The accurate diagnosis of the malignant ascites primary origin is critical for planning an effective treatment and practically predicting the prognosis. In this retrospective study, we have demonstrated that regional delayed ^18^F-FDG PET/MRI-assisted whole-body ^18^F-FDG PET/CT significantly improves the diagnosis of malignant ascites. The primary tumor on PET/MRI appears more prominent, which enhances diagnostic accuracy. Additionally, PET/MRI improved the detection of metastatic lesions comparing to PET/CT alone.

Some previous studies have reported that for cancer TNM staging and diagnostic accuracy, ^18^F-FDG PET/CT and PET/MRI perform equally well (Spick et al. 2016; Czernin et al. 2014). However, based on our results, the diagnostic accuracy of regional PET/MRI-assisted whole-body PET/CT is much higher than PET/CT alone in identifying the primary tumor of malignant ascites. In the visual assessment of the primary tumor, the PET/MRI had a higher conspicuity score comparing to PET/CT, giving physicians increased confidence with improved diagnostic accuracy, especially in ovarian cancer, which is the most common type of primary cancer associated with ascites and accounts for 38% of malignant ascites in females (Kipps et al. 2013). In our study, the detection rate of ovarian cancer by PET/MRI was 100% (11/11). With the assistance of PET/MRI, we were able to correctly diagnose 7 more cases of gynecological malignant tumors. This was made possible mainly due to the high soft tissue resolution and clear anatomical display of MR imaging. MRI has the advantages of excellent soft tissue contrast, superior spatial resolution, and functional sequences such as diffusion-weighted imaging (DWI), which can show anatomical details and component of the lesions more clearly than CT. In CT of PET/CT, the CT attenuation of primary ovarian tumor is not distinguishable from multiple peritoneal nodules. However, in MRI of PET/MRI, ovarian tumors typically exhibit cystic or polycystic components, different from the solid component nodules of peritoneal metastases, which helps to distinguish the primary lesion from multiple peritoneal metastatic nodules.

Unfortunately, despite the assistance of PET/MRI, 3 cases of peritoneal carcinoma were still misdiagnosed as ovarian and colon cancer. The diagnostic accuracy for primary peritoneal cancer is the lowest, because it is often diagnosed when there is no exact primary tumor, and metastatic nodules or masses in the abdominal cavity can be misinterpreted as primary malignancies in adjacent organs. Primary peritoneal serous carcinoma is histologically identical to ovarian serous carcinoma and may be indistinguishable from metastatic ovarian carcinoma in imaging (Levy et al. 2008).

In terms of metastases, PET/MRI identifies more metastases than PET/CT, which is important for the M staging. Multiparametric MR imaging has advantages in detecting small lesions. The metastases lesions in parenchymal organ and bone marrow, which have hyperintensity on T2-weighted image, are more remarkable than on CT images. Additionally, DWI makes the detection of metastasis highly sensitive (Fujii et al. 2008; Low et al. 2009). According to a meta-analysis, the sensitivity and specificity estimates for MRI in detecting bone lesions were 90.4% and 96.0%, about 15% higher than CT (Yang et al. 2011). Additionally, more parenchymal organ metastases, including liver, spleen metastases, were identified in PET/MRI than in PET/CT. As reported, the diagnostic value of PET/MRI for hepatic metastases is higher than PET/CT (Zhou et al. 2021; Yong et al. 2011). For lesions smaller than 10 mm, the detection sensitivity for MR imaging was higher than CT (Niekel et al. 2010). In our study, the smallest metastasis, a 4 mm in diameter of liver metastases, was detected by PET/MRI, while it was missed in PET/CT. PET/MRI has also identified more peritoneal metastases and metastatic lymph nodes. Radiologist sensitivity and specificity of MRI, especially DWI, on detecting peritoneal metastases have been reported to be as high as 90% or more (Fujii et al. 2008; Low et al. 2009). And studies have shown that PET/MRI is equal to or superior to PET/CT for lymph node detection (Beiderwellen et al. 2015; Kim et al. 2009). Moreover, in our study, delayed PET also plays a role in improving the detection of lesions. The lesion-to-background ratio of SUVmax is more evident due to progressive accumulation of FDG tracers over time (Chan et al. 2011; Drzezga et al. 2012), which makes the lesions more prominent in delayed PET/MRI than in PET/CT and helps to improve the detection of small lesions. In our study, partial lesions, especially peritoneal metastases, have been detected due to the accumulation of FDG in delayed PET, rather than MRI.

Due to its time-consuming processes and cost considerations, whole-body PET/MRI is not widely used in clinical practice at present. Currently, there are several abdominal PET protocols for diagnosing abdominal tumors, including additional CT or MR scans combined with whole-body PET/CT, delayed abdominal PET/CT or PET/MRI scan after whole-body PET/CT (Schwenzer et al. 2012). Our examination protocol includes a delayed regional PET/MRI scan after the completion of whole-body PET/CT. This one-stop-for-all hybrid PET/MRI scan provides excellent anatomical information of MR and synchronous metabolic information of PET without additional ionizing radiation, which is more efficient than CT or MR examinations alone. Moreover, this protocol takes less time than whole-body PET/MRI, which takes about 10–30 min for regional pelvic and abdominal PET/MRI, while the whole-body PET/MRI takes more than an hour. Meanwhile, the cost of regional PET/MRI scan is approximately one-third of whole-body scan. Therefore, delayed regional PET/MRI-assisted whole-body PET/CT protocol can not only improve the diagnostic efficiency, but also shorten the examination time and reduce the cost comparing to whole-body PET/MRI, which is beneficial for patients with massive ascites and difficult to lie flat for a long time. However, the scan range of regional PET/MRI depends on the prior whole-body PET/CT findings and the doctor's initial judgment. In 1 case of our study, the PET/MRI scan range was inadequate to include the primary lesion, resulting in an incorrect diagnosis.

There are some limitations of our research. Firstly, this study is limited by its retrospective nature with inevitable selection bias, the sample size of this study is statistically small, and most cases are female patients. Secondly, this study is not a strict head-to-head comparison of PETMRI and PETCT, the delayed PET also plays a role in improving diagnostic accuracy. Nevertheless, additional regional PET/MRI imaging clearly showed advantages for the diagnosis of primary tumor for malignant ascites, which may be contribute for clinical decision-making. More prospective cases will be included in future to further validation this modality.

## Conclusion

Comparing to whole-body PET/CT alone, a combined delayed regional PET/MRI proves to be more effective in diagnosing the location of primary tumor and detecting metastases. In assessment of the primary tumor, the additional PET/MRI lesions, especially ovarian cancer, were mainly attributed to MRI, while, in terms of metastases, both delayed PET and MRI play roles in improving the detection.

## Data Availability

The datasets used and/or analyzed during the current study are available from the corresponding author on reasonable request.

## Abbreviations

FDG: Fluorodeoxyglucose
PET: Positron emission tomography
MR: Magnetic resonance
CT: Computed tomography
SUVmax: Maximum standard uptake value
DWI: Diffusion-weighted imaging
ADC: Apparent diffusion coefficient
